# Protein swarm-based cause-effect analysis: effects of microRNAs on cooperation networks linking COVID-19 infections, atherosclerosis, and Alzheimer's disease

**DOI:** 10.3389/fcvm.2025.1577844

**Published:** 2025-12-08

**Authors:** Anton Fliri, Ron Sostek, Shama Kajiji

**Affiliations:** 1 SystaMedic Inc., Clinton, CT, United States; 2 Emergent System Analytics, LLC., Clinton, CT, United States

**Keywords:** material protein swarms, gene ontology, proteomics, atherosclerosis, microRNAs, infections, Alzheimer's disease, cardiovascular conditions

## Abstract

Well-being depends on the integrated operation of biological processes at all levels of system organization, from individual cells to tissues and organ systems, collectively sustaining homeostasis and optimal bodily functions. The regulation of cooperation among these processes is mediated by information flow within networks possessing diverse structural, functional, and temporal properties. Disruption in these networks is observed in conditions such as infections, inflammatory diseases, and cancer. To advance understanding of immune system roles and to elucidate mechanisms underlying health vulnerability during disease, we utilized proteomics data related to 4,800 diseases along with protein swarm-based cause-effect analyses to identify principles governing plasticity and self-organizing capabilities of immune systems. Our findings demonstrate that the precision of immune system functions is regulated by dynamic alterations in the topologies of cooperation networks that are partially modulated by microRNAs. Additionally, our analysis indicates that investigating the underlying causes of diseases through the study of cooperative network functions and their interactions with microRNAs—rather than concentrating exclusively on individual protein targets or microRNAs—provides significant insights for devising effective treatment strategies for infections, cardiovascular conditions, Alzheimer's disease, cancer, aging, and related health concerns.

## Introduction

Swarm intelligence is widely recognized as a key factor in the remarkable precision of immune systems, and this emergent property is now being simulated through computer (1, 2) Immune systems have evolved to protect organisms against a broad spectrum of challenges, including cancer, harmful substances, damaged proteins, DNA, RNA, and infections caused by viruses, bacteria, fungi, and parasites (3–5). Key functions of the innate immune system include rapid elimination of various pathogens (6, 7), development of immunological memory to prevent reinfection (8), avoiding miscarriage and maintaining pregnancy (9), supporting beneficial relationships between the host and microbiome (10), and minimizing the risk of autoimmune disease (11).

The role of the innate immune system extends beyond these core functions to regulation of tissue homeostasis (12), metabolism (13), stem cell differentiation (14), the regulation of interactions between organ systems (15), development of the central nervous system (16), and the persistence of memory and cognition (17, 18). These diverse actions rely on immune cells that display plasticity and adapt their behavior depending on injuries in a macro and micro environment-specific manner (19–21). The rules regulating this adaptability involve epigenetic regulation at all system levels (22–24). Dysregulation of these processes can occur in the context of infections (25–31), cardiovascular diseases (32, 33), dementias (34, 35), metabolic diseases (36, 37), and cancer (38, 39). The complexity and interdependence (cause-effect relationships) of these dynamic systems create significant challenges in the development of safe and effective disease treatment and prevention options (40–44).

Addressing these challenges requires an understanding of the cause-effect relationships involving interactions of multimode cooperation and communication networks with dynamic network topologies (45–55). Because variables governing dynamic network-network interaction in biological networks are often unknown, we have developed methods—such as protein swarm analysis and cause-effect analysis – to capture plasticity and emergent properties of these complex dynamic interaction systems (56). By integrating concepts from pharmacological cause-effect analysis (57), information theory (51), communication networks (47), cooperation networks (58), and particle swarm optimization (53, 59, 60), protein swarm-based cause- effect analysis determines variations in information exchange among protein swarms to identify network structures associated with discrete system perturbations (50, 57, 61–63). This analytical approach (cause-effect linkage) organizes information within large, orthogonal, decentralized data sets and sets constraints for solutions to global network architectures that generate emergent properties (64). This method aims to be robust against local data gaps or inaccuracies and provides global, unbiased insight into complex cause-effect relationships (65–67).

Given recent findings that COVID-19 may be associated with negative cardiovascular outcomes (such as heart failure and acute coronary syndromes), and that bidirectional relationships exist among atherosclerosis, Alzheimer's disease, and COVID-19 (68, 69), this study applies cause-effect analysis to investigate how COVID-19 influences regulatory rules in immune system function across 4,800 diseases—including cardiovascular diseases, dementias, 80 types of cancer, and over 350 different infectious diseases (70).

## Materials

Protein swarms are generated by dividing tissue and cell-associated protein expression data published in the Human Protein Atlas (71), into information transfer modules using the String platform's gene enrichment analysis for identifying overlaps between tissue proteomes and biological processes (Gene ontology) (72). Proteins captured in this network overlap analysis containing no more than 5 proteins are collected and labeled for identifying tissue and functional associations (73). Using 7,323 of these protein swarms and a data mining tool developed by SystaMedic Inc. in collaboration with the University of Connecticut enabled the determination of co-citation frequencies of these 7,323 protein swarms with 4,800 diseases in millions of PubMed abstracts (74). Viewing collected co-citation frequency measurements as indicators of the capacity of a wide range of system perturbations to impact information exchanges between protein swarms, we collected 7,323 × 4,800 of these measurements and determined similarities between the resulting information spectra using hierarchical clustering in Spotfire (75–77). The outcome of this analysis produced an equilibrium network structure organizing 7,323 protein swarms and 4,800 diseases (56, 57, 78–84). The translation of protein swarm information into protein network structures and retrieval of pre-existing knowledge associated with protein networks used the STRING platform (85, 86).

## Methods

**Figure 1:**
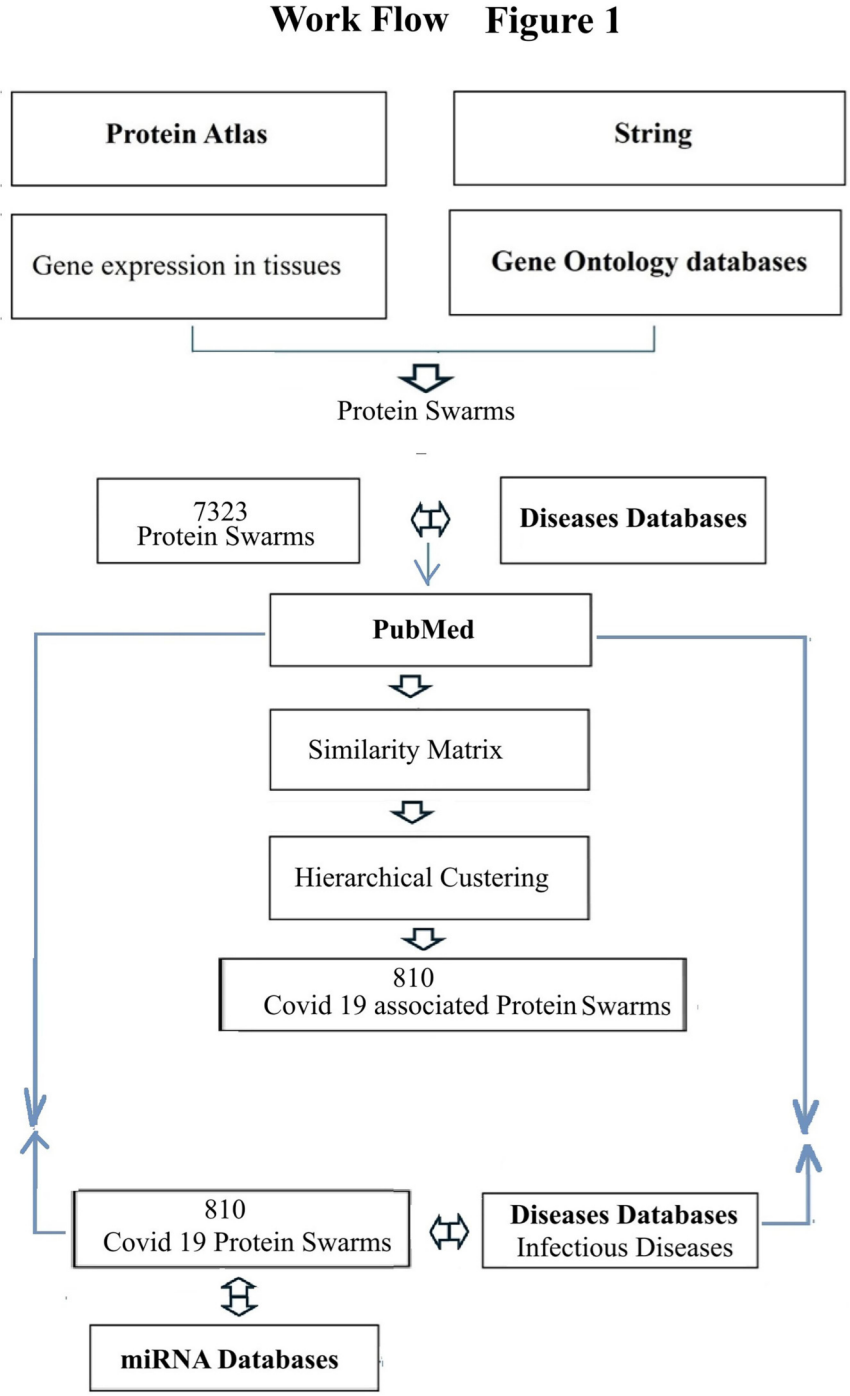
Process map of steps taken for swarm-based, cause-effect analsyis. Bolded text indicate the main databases that were used.

To identify rules regulating immune system functions in over 4,800 diseases, we selected COVID-19-associated proteomics and microRNA expression data generated during the recent COVID-19 pandemic (87, 88), and accessible via PubMed (89), HMDD (90), and miRBase (91) as a starting point for this investigation (Figure 1).

## Terminology

The term ***cooperation*** refers to working together on accomplishing specific tasks or solving problems. The term ***coordination*** refers to the process of organizing and integrating distinct functions, making sure that distinct parts of a system are working together as needed to produce desired outcomes. The term ***disease-associated proteomics information*** refers to data describing proteins associated with a disease, how they are modified, and how they interact. The term ***emergent property*** refers to properties that arise when interactions between individual components produce new functions. The term ***network topology*** refers to the way different nodes in a network are positioned and interconnected, as well as how information flows. The term ***dynamic network topology*** refers to a network structure where nodes can join or leave the network, and information transmission can adapt to changing needs or conditions. The term ***network overlap analysis*** refers to the process of identifying shared nodes within networks. The term ***plasticity*** refers to the ability of the immune system to change its phenotype or function in response to environmental stimuli. The term ***protein swarm*** refers to a collection of proteins capable of interacting with other proteins and participating in biological functions involving these protein interactions. The term ***self-organization capacity of proteins*** refers to the emergence of an overall order in time and space resulting from the collective interactions of individual proteins. The term ***spectral clustering*** refers to a machine learning technique that groups data points into clusters by analyzing the connectivity between them using values of a similarity matrix.

## Results

### Spectral clustering

**Figure 2:**
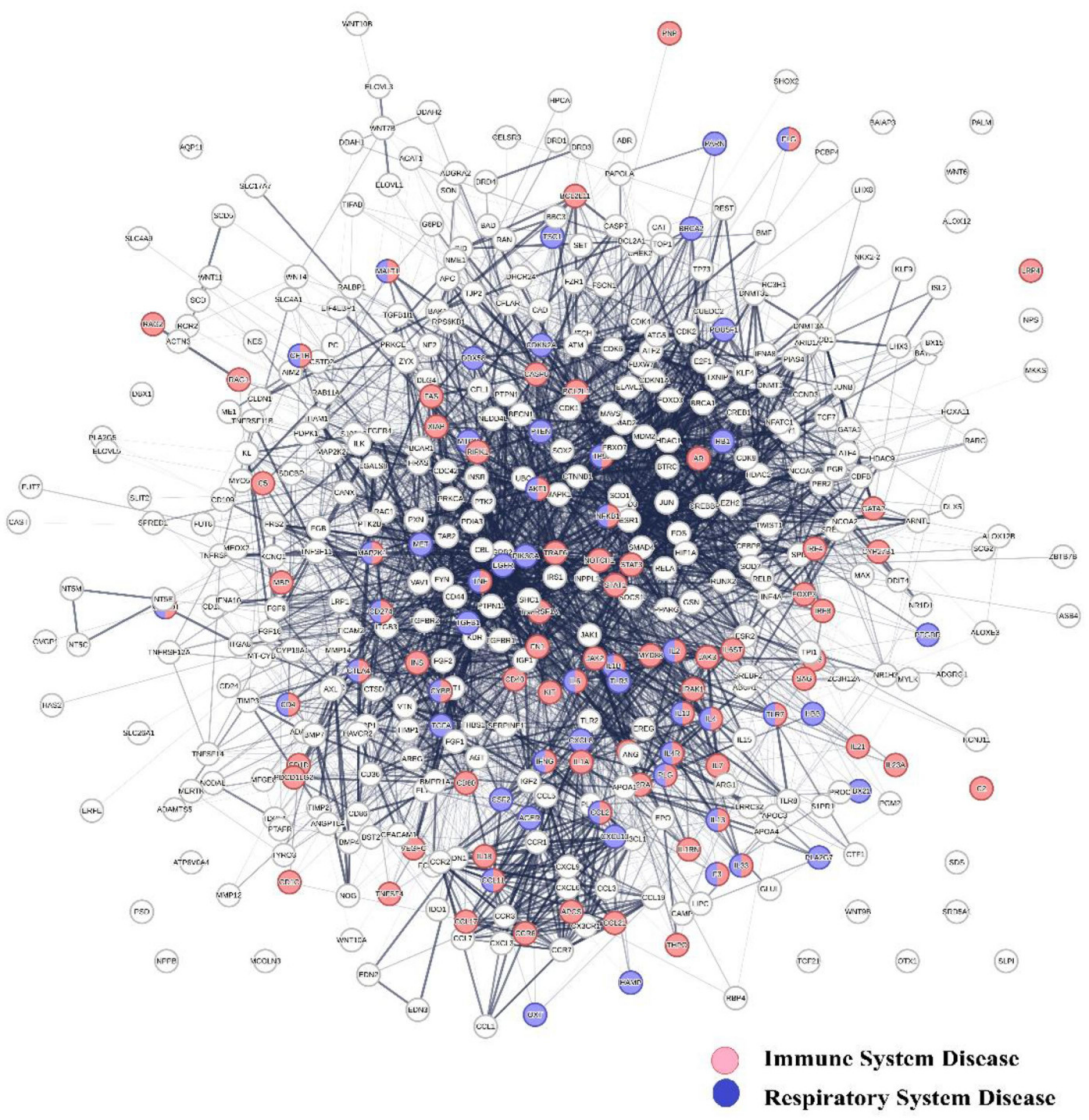
Shows the physical interaction between 444 host proteins associated with COVID 19 and identifies network overlaps between immune system diseases (red network nodes) and respiratory system diseases (blue nodes).

The hierarchical clustering of co-citation frequency measurements for 4,800 diseases and 7,323 protein swarms has been described in a previous publication (94). Building upon the observation of a notable association between COVID-19 infections and cardiovascular disease, this study utilized this published analysis to identify COVID-19-associated proteomics data, applying a cluster similarity confidence value (CCSV) threshold greater than 0.92. As outlined in Supplementary Table S1 of the Supplemental section, the analysis revealed 33 protein swarm associations (PSA), encompassing a total of 810 protein swarms and 444 host proteins. A network-based perspective demonstrates in Figure 2 that these 444 host proteins exhibit potential for physical interaction. Furthermore, network overlaps analysis indicates that COVID-19 affects host proteins integral to immune system disorders (false discovery rate: 2.14 × 10^−30^) (92), and respiratory disease (false discovery rate: 9.82 × 10^−27^) (93, 94). These results provide evidence that COVID-19 induces profound system-wide disturbances of immune system functions (95–98).

**Figure 3:**
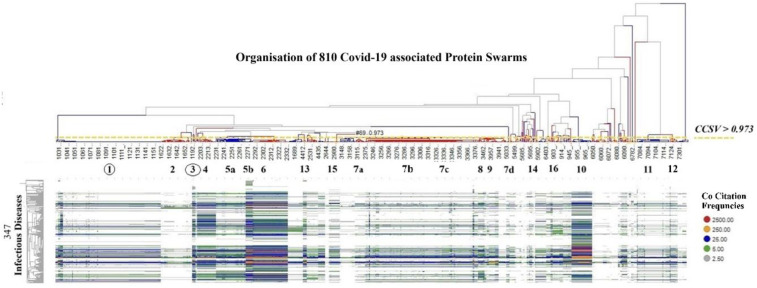
Shows the alignment of proteomics information associated with 347 infectious diseases, Vitamin D deficiency, Alzheimer's disease, and atherosclerosis, using as comparative standard 20 protein swarm clusters associated within confidence in cluster similarity values of >0.973. This high confidence in cluster similarity value shows that infectious diseases, vitamin D deficiency, Alzheimer's disease and atherosclerosis have closely aligned proteomics information in the highlighted protein swarm clusters. Red identifies the highest and grey the lowest number of co-citation frequencies.

To evaluate the potential of COVID-19-associated proteomics data as a basis for identifying regulatory mechanisms governing system-wide immune system functions (99, 100), we analyzed co-citation frequencies of COVID-19-associated protein swarms listed in Supplementary Table S1 and 4,800 diseases described in PubMed abstracts. Hierarchical clustering of the resulting 810 × 4,800 information spectra matrix revealed alignments between COVID-19-associated proteomics profiles and those associated with these 4,800 diseases. To enable effective visual data comparison, we applied a cluster similarity confidence threshold of >0.973 for segmenting and dividing COVID-19-associated proteomics information into 20 discrete clusters. Examination of co-citation frequency measurements within these clusters indicated that 1,276 diseases—including Alzheimer's disease, 38 cancer types, 85 carcinomas, 157 inflammatory conditions, and 347 infections—demonstrated close proteomics data alignment within these sections. This finding is illustrated in Figure 3 which shows the alignment of proteomics information associated with 347 infectious diseases, vitamin D deficiency (101), Alzheimer's disease (102), and atherosclerosis (103, 104). The specific names of the infectious diseases referenced in Figure 3 can be found in Table 1 of the supplemental section.

**Table 1:** List of diseases having closely-aligned proteomics information with COVID-19.

Acinetobacter baumannii pneumonia	Diphtheria	Japanese encephalitis	Paracoccidioidomycosis	Severe acute respiratory syndrome
Acquired immunodeficiency syndrome	Dysentery	Japanese spotted fever	Paragonimiasis	Severe combined immunodeficiency
Acquired thrombocytopenia	Early congenital syphilis	Kaposi's sarcoma	Paratuberculosis	Smallpox
Adenovirus pneumonia	Eastern equine encephalitis	Kawasaki disease	Paratyphoid fever	Sporotrichosis
AIDS-related Kaposi's sarcoma	Ehrlichiosis	Keratitis	Paronychia	Spotted fever
Alveolar echinococcosis	Encephalitis	Klebsiella pneumonia	Pasteurellosis	St. Louis encephalitis
Amebic colitis	Endemic typhus	Laryngeal tuberculosis	Pemphigus	Staphylococcal pneumonia
Avian influenza	Eosinophilic meningitis	Lassa fever	Pericarditis	Staphylococcus aureus septicemia
Avian malaria	Eosinophilic pneumonia	Latent syphilis	Periodontitis	Streptococcal necrotizing fasciitis
Babesiosis	Ephemeral fever	Legionellosis	Peritonitis	Streptococcal pharyngitis
Bacterial conjunctivitis	Equine encephalitis	Legionnaires' disease	Pertussis	Streptococcal pneumonia
Bacterial endocarditis	Erysipelas	Leishmaniasis	Pharyngitis	Streptococcal septicemia
Bacterial gastroenteritis	Erythema infectiosum	Lepromatous leprosy	Plague	Subacute bacterial endocarditis
Bacterial infectious disease	Extrapulmonary tuberculosis	Leprosy	Plasmodium falciparum malaria	Swine influenza
Bacterial meningitis	Fascioliasis	Leptospirosis	Plasmodium vivax malaria	Syphilis
Bacterial pneumonia	Feline infectious peritonitis	Listeria meningitis	Pleural tuberculosis	Systemic inflammatory response syndrome
Bacterial prostatitis	Female genital tuberculosis	Listeriosis	Pneumatosis cystoides intestinalis	Systemic mycosis
Bacterial vaginosis	Filariasis	Lobomycosis	Pneumococcal meningitis	Takayasu's arteritis
Botulism	Folliculitis	Louse-borne relapsing fever	Pneumococcal pneumonia	Tetanus
Bovine tuberculosis	Fowlpox	Lyme disease	Pneumoconiosis	Tick-borne encephalitis
Bubonic plague	Fungal meningitis	Lymphocytic choriomeningitis	Pneumocystosis	Tinea
Campylobacteriosis	Fusariosis	Malaria	Pneumonic pasteurellosis	Toxic shock syndrome
Central nervous system tuberculosis	Genital herpes	Mastitis	Podoconiosis	Toxoplasma myocarditis
Cerebral malaria	Giardiasis	Measles	Polioencephalitis	Toxoplasmosis
Cerebral toxoplasmosis	Glossitis	Melioidosis	Poliomyelitis	Trichinosis
Chagas cardiomyopathy	Gonococcal urethritis	Meningitis	Polycythemia vera	Trichomonas vaginitis
Chagas disease	Gonorrhea	Meningococcal meningitis	Polymyositis	Trichomoniasis
Chickenpox	Haemophilus influenzae meningitis	Meningoencephalitis	Pontiac fever	Trichuriasis
Chronic fatigue syndrome	Helicobacter pylori gastritis	Microsporidiosis	Porcine reproductive and respiratory syndrome	Trypanosomiasis
Chuvash polycythemia	Hemorrhagic disease	Mixed malaria	Primary amebic meningoencephalitis	Tuberculosis
Classic Kaposi's sarcoma	Hepatic encephalopathy	Molluscum contagiosum	Primary syphilis	Tularemia
Classical swine fever	Hepatitis A	Monkeypox	Primary tuberculosis	Tungiasis
Coccidioidomycosis	Hepatitis B	Mouth disease	Prion disease	Typhoid fever
Coccidiosis	Hepatitis C	Mucocutaneous leishmaniasis	Pseudorabies	Typhus
Colitis	Hepatitis D	Mucormycosis	Pulmonary cryptococcosis	Ulcerative colitis
Colorado tick fever	Hepatitis E	Multidrug-resistant tuberculosis	Pulmonary mucormycosis	Vaccinia
Congenital rubella	Herpes simplex	Mumps	Pulmonary sporotrichosis	Variant Creutzfeldt-Jakob disease
Congenital syphilis	Herpes simplex virus encephalitis	Mycoplasma pneumoniae pneumonia	Pulmonary tuberculosis	Viral encephalitis
Conjunctivitis	Histoplasmosis	Mycoplasmal pneumonia	Pyelonephritis	Viral gastroenteritis
Coronavirus infectious disease	HIV encephalopathy	Mycosis fungoides	Q fever	Viral hepatitis
COVID 19	HIV-associated lipodystrophy syndrome	Myositis	Rabies	Viral infectious disease
Cowpox	HIV-associated nephropathy	Neuritis	Respiratory syncytial virus bronchiolitis	Viral meningitis
Crimean-Congo hemorrhagic fever	Human monocytic ehrlichiosis	Newcastle disease	Respiratory syncytial virus pneumonia	Viral pneumonia
Cryptococcal meningitis	Indian tick typhus	Norovirus gastroenteritis	Rhinocerebral mucormycosis	Vitamin D deficiency
Cryptococcosis	Infectious canine hepatitis	Ocular toxoplasmosis	Rhinosporidiosis	Vulvovaginal candidiasis
Cryptosporidiosis	Infectious mononucleosis	Ocular tuberculosis	Rickets	West Nile fever
Cyclosporiasis	Infective endocarditis	Omsk hemorrhagic fever	Rift Valley fever	Western equine encephalitis
Cystic echinococcosis	Inflammatory bowel disease	Onchocerciasis	Rocky Mountain spotted fever	Wuchereria bancrofti filariasis
Cystoisosporiasis	Influenza	Onychomycosis	Rotavirus gastroenteritis	Wound botulism
Cytomegalovirus colitis	Influenza encephalopathy	Opisthorchiasis	Rubella	Xeroderma pigmentosum
Cytomegalovirus pneumonia	Intermediate uveitis	Oral candidiasis	Salmonella gastroenteritis	Yellow fever
Dengue disease	Intestinal schistosomiasis	Oral tuberculosis	Salpingitis	Zika fever
Dengue hemorrhagic fever	Intestinal tuberculosis	Orchitis	Scabies	SARS-CoV-2 spike protein S1
Dengue shock syndrome	Invasive aspergillosis	Oropharyngeal candidiasis	Scarlet fever	SARS-CoV-2 nucleocapsid protein
Dermatomycosis	Iridocyclitis	Osteomyelitis	Schistosomiasis	COVID-19 vaccine

### Identification of protein networks involved in immune responses

To identify the roles of protein interactions within protein swarm associations **1–20** in Figure 3 for immune responses, Figure 2 was used for protein network overlap analysis (105). Using a false discovery rate of 5.42 × 10^−5^ in the Kegg database (106), and 3.94 × 10^−9^ in the Gene Ontology database (107), we identified 94 different immune system functions involving proteins in these protein swarm associations. These functions are listed in Table 2 of the supplemental section.

**Table 2:** List of biological processes involved in immune responses.

Adipocytokine signaling pathway	mTOR signaling pathway
AGE-RAGE signaling pathway in diabetic complications	Myeloid leukocyte activation
Aldosterone-regulated sodium reabsorption	Myeloid leukocyte differentiation
Allograft rejection	Myeloid leukocyte migration
AMPK signaling pathway	Natural killer cell mediated cytotoxicity
Apelin signaling pathway	Negative regulation of T cell activation
Apoptosis	Neurotrophin signaling pathway
Axon guidance	Neutrophil chemotaxis
B cell receptor signaling pathway	NOD-like receptor signaling pathway
Cell cycle	p53 signaling pathway
Cellular response to interferon-gamma	Pathways in cancer
Cellular response to virus	Positive regulation of angiogenesis
Cellular senescence	Positive regulation of ERK1 and ERK2 cascade
cGMP-PKG signaling pathway	Positive regulation of leukocyte chemotaxis
Chemokine signaling pathway	Positive regulation of MAP kinase activity
Chemokine-mediated signaling pathway	PPAR signaling pathway
Cholesterol metabolism	Protein kinase B signaling
C-type lectin receptor signaling pathway	Ras signaling pathway
Cytokine-cytokine receptor interaction	Regulation of actin cytoskeleton
Cytosolic DNA-sensing pathway	Regulation of chemokine production
Dendritic cell differentiation	Regulation of endothelial cell migration
EGFR tyrosine kinase inhibitor resistance	Regulation of epithelial to mesenchymal transition
Endoderm development	Regulation of glial cell differentiation
Epidermal growth factor receptor signaling pathway	Regulation of gliogenesis
ERBB signaling pathway	Regulation of immune system process
ErbB signaling pathway	Regulation of leukocyte chemotaxis
Estrogen signaling pathway	Regulation of osteoblast differentiation
Fc epsilon RI signaling pathway	Regulation of T cell differentiation
Fc gamma R-mediated phagocytosis	Regulation of T cell proliferation
FoxO signaling pathway	Relaxin signaling pathway
Genitalia development	Renal tubule development
Glial cell activation	Response to interferon-gamma
GnRH signaling pathway	Response to lipopolysaccharide
Granulocyte chemotaxis	Response to molecule of bacterial origin
HIF-1 signaling pathway	Response to stress
Hippo signaling pathway	RIG-I-like receptor signaling pathway
IL-17 signaling pathway	Signaling pathways regulating pluripotency of stem cells
Intestinal immune network for IgA production	Sphingolipid signaling pathway
JAK-STAT signaling pathway	T cell differentiation
Leukocyte chemotaxis	T cell receptor signaling pathway
Longevity regulating pathway	TGF-beta signaling pathway
Macrophage activation	Th1 and Th2 cell differentiation
Macrophage Polarization	Th17 cell differentiation
MAPK signaling pathway	Toll-like receptor signaling pathway
Microglia Polarization	Ubiquitin mediated proteolysis
Monocyte chemotaxis	Viral carcinogenesis
Mononuclear cell migration	Wnt signaling pathway

**Figure 4:**
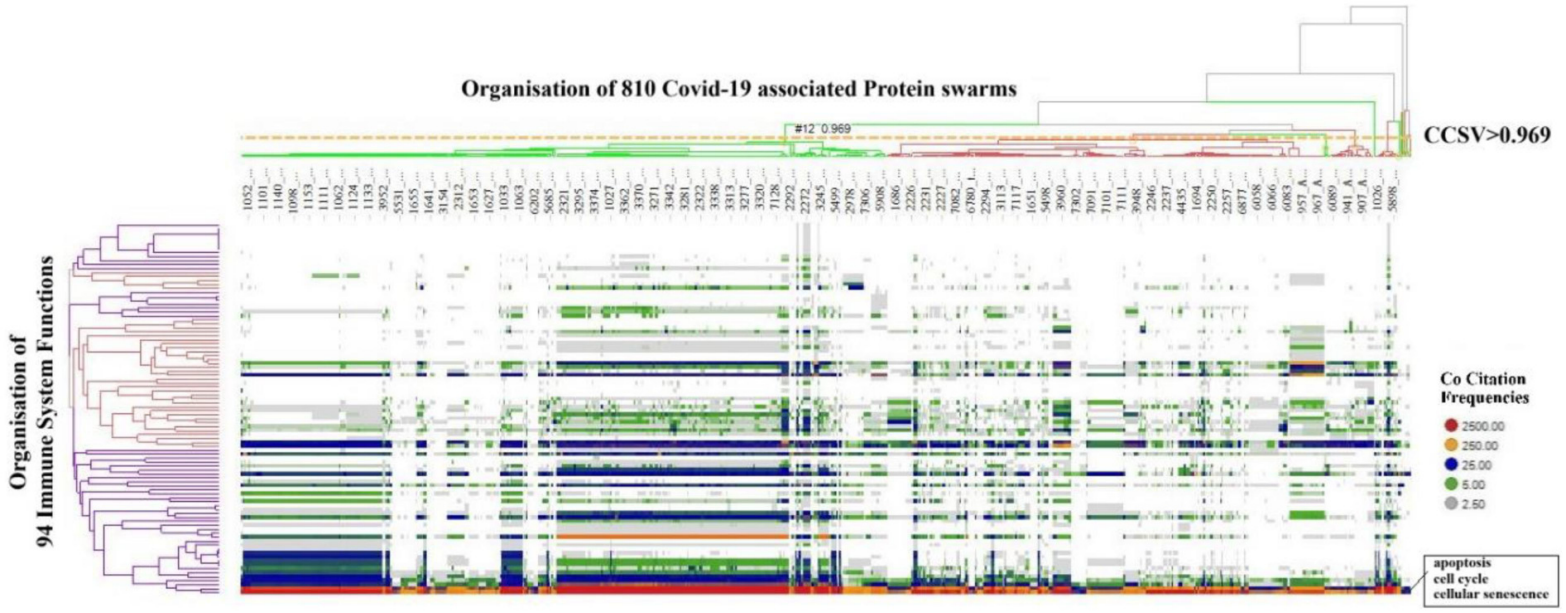
Identifies alignment of proteomics information associated with COVID 19 and proteomics information associated with 94 biological functions involved in host responses to infections.

To determine functional relationships between protein networks among these immune functions, co-citation frequencies of COVID-19-associated protein swarms with the 94 immune functions were calculated from Medline abstracts. The result of the hierarchical clustering of these data, presented in Figure 4, shows that protein swarm associations **1–20,** linked within confidence in cluster similarity values greater than 0.969, divide the 94 immune system functions into discrete clusters. Additionally, the characteristic distribution of proteomics information in these clusters indicates that the 94 immune functions and protein swarm associations **1–20** in Figure 3 coregulate immune responses in 347 infections, vitamin D deficiency, Alzheimer's disease, and atherosclerosis (108–110).

### Impact of disease-associated microRNAs on host responses to infections

**Figure 5:**
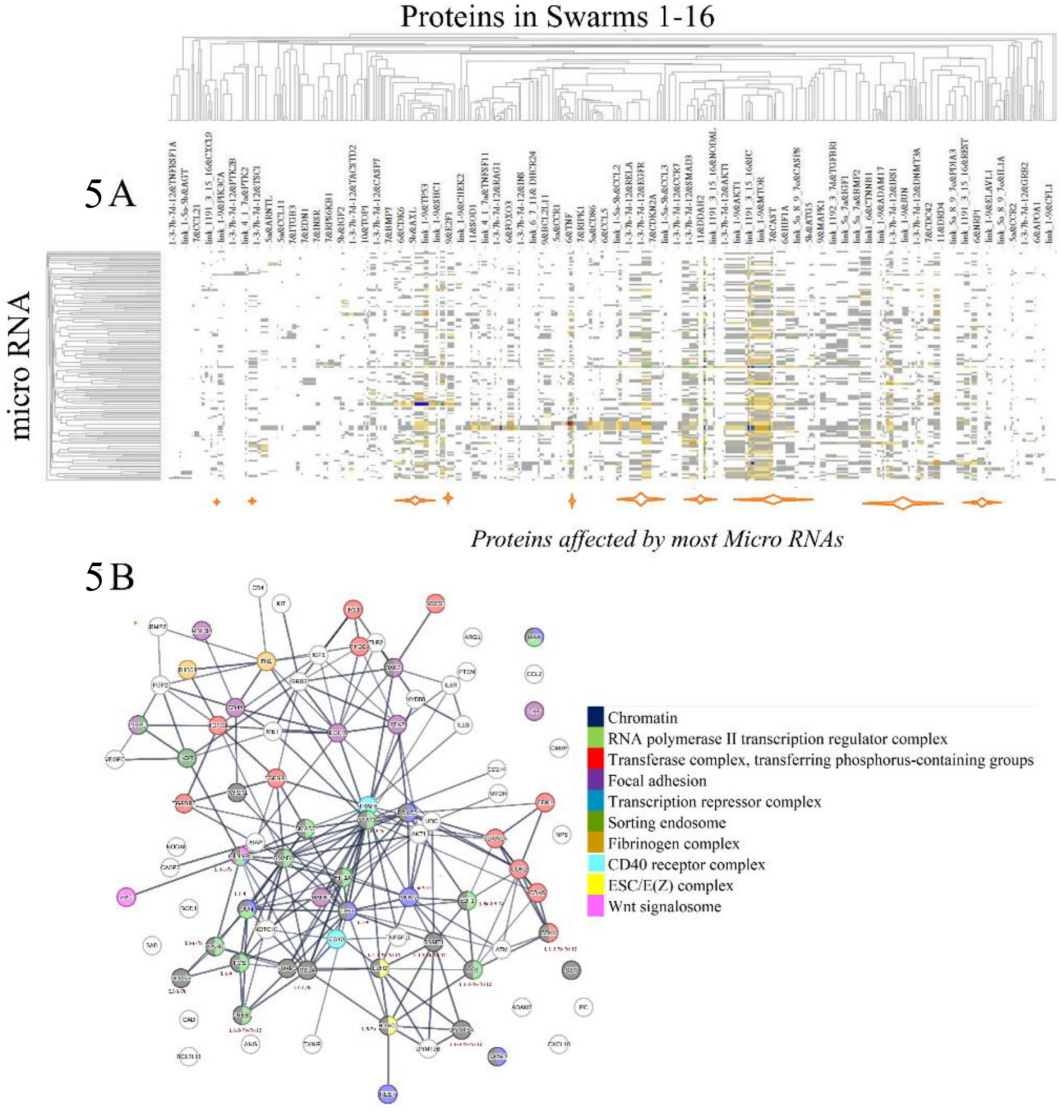
**(A)** identifies the effects of disease-associated microRNAs on proteins constituting protein swarm associations 1-20. Orange bars at the bottom of **(A)** identify the positions of proteins targeted by most disease-associated microRNAs. **(B)** Identifies physical interactions between proteins targeted by disease-associated microRNA. Proteins highlighted in colors are members of protein complexes located in an intracellular membrane-bounded organelle known to be targeted in infections.

To investigate the effects of disease-associated microRNAs on protein network-network interactions as depicted in Figure 4 (111), we applied two distinct methodologies. The first method aimed to identify functional relationships among proteins targeted by disease-associated microRNAs. Specifically, we examined proteins captured in protein swarm associations **1–20** and determined their co-citation frequencies with 136 mature microRNAs associated with COVID-19 and Alzheimer's Disease in Medline abstracts (112–115). Hierarchical clustering of the resulting similarity matrix visualized in the heatmap in Figure 5A indicates that disease-associated microRNAs—particularly mir-155, mir-146a, mir-34a, and mir-21, which are recognized for their role in immune system functions (116–119), Alzheimer's disease (120–123), and atherosclerosis (124–127),—regulate expression levels of all proteins captured in protein swarm associations **1–20**. From a protein network perspective, as shown in Figure 5B, these microRNAs alter the expression levels of proteins located within intracellular membrane-bound organelles (128), and these proteins participate in complexes that are critical to immune responses.

For example, chromatin is implicated at the intersections of viral infections and DNA damage control (129), as well as Alzheimer's disease (130) atherosclerosis (131). The RNA polymerase II transcription regulator complex and the transferase complex transferring phosphorus-containing groups play key roles in influenza infections (132, 133), Alzheimer's disease (134), and atherosclerosis (135).

Regulation of focal adhesion is the target of pathogenic microbes (136), contributes to cell death in Alzheimer's disease (137), and is involved in the progression of atherosclerosis (138). The transcription repressor complex plays a key role in RNA virus infections (139), Alzheimer's disease (140), and atherosclerosis (141). Sorting endosome functions are hijacked in viral infections (142), Alzheimer's disease (143), and atherosclerosis (144), while the fibrinogen complex functions are targeted in bacterial infections (145), Alzheimer's disease (146), and atherosclerosis (147).

Functions of the CD40 receptor complex are central to immunity (148), Alzheimer's disease (149), and atherosclerosis (150), and the ESC/E(Z) complex governs the expression of immune-related genes (151, 152), participates in Alzheimer's disease (153), and atherosclerosis (154). Furthermore, the Wnt signalosome is pivotal in host responses to infections (155), Alzheimer's disease (156), and atherosclerosis (157). These findings indicate that protein swarm associations **1–20** coordinate functions of various protein complexes, and that microRNAs such as mir-155, mir-146a, mir-34a, and mir-21, influence the expression levels of proteins within these protein swarm associations **1–20**, thereby, affecting the topology of networks that regulate functions of protein-machinery involved in immune responses to injuries and infections (158). Consequently, protein target-focused cause-effect analysis provides insight into the proximities of microRNA targets within protein networks (159), the cellular localization, and the functions of cellular machinery influenced by microRNAs (160). Additionally, proteins within swarm associations **1–20** are components of protein complexes engaged in multiple immune functions. However, due to the extensive array of proteins targeted by microRNAs, establishing unambiguous regulatory relationships between the 136 analyzed microRNAs and the proteins in swarm associations **1–20** is not feasible (161–163).

**Figure 6:**
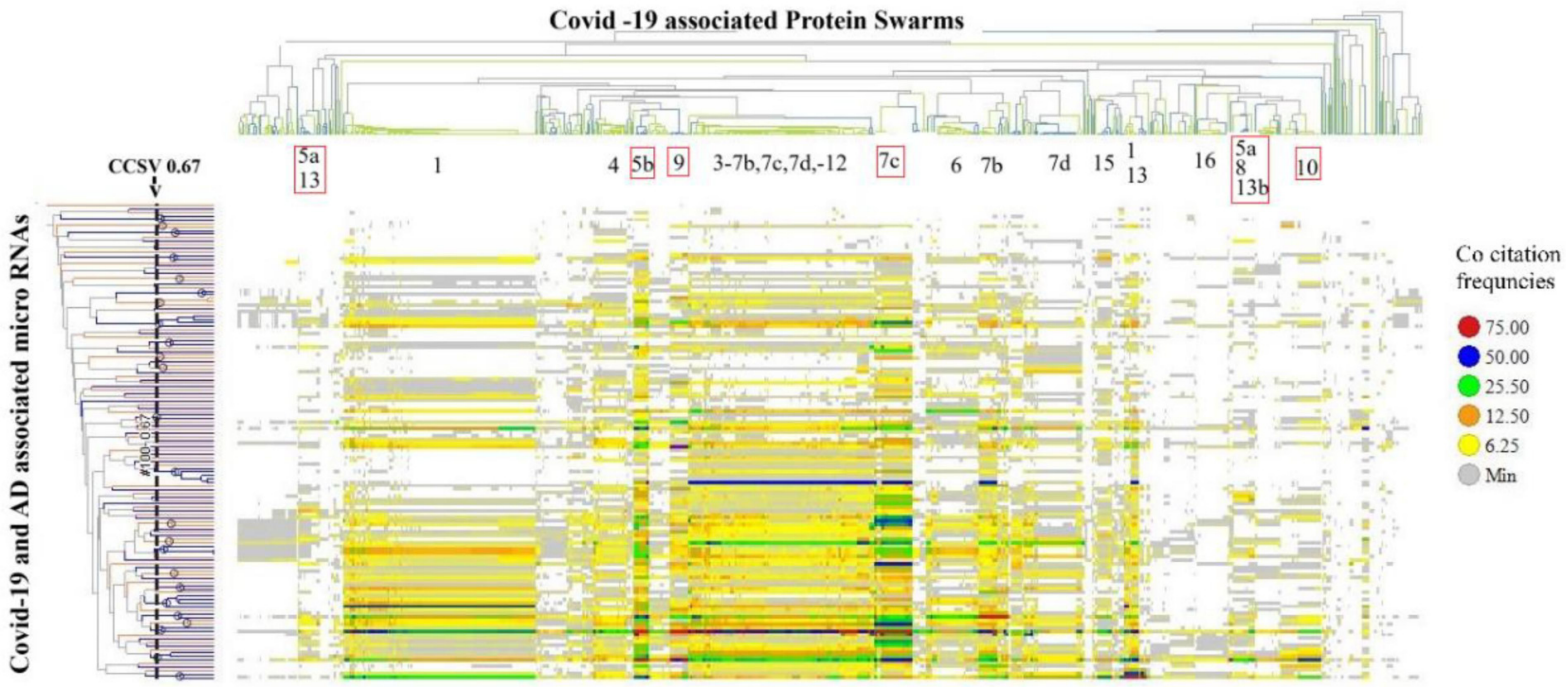
Identifies protein swarm associations (**1–16**) collected within a confidence in cluster similarity value of >0.897 and 23 microRNA clusters collected within a confidence in cluster similarity value of >0.67.

The second method for evaluating the impacts of disease-associated microRNAs on protein swarm associations **1–20** (as depicted in Figure 3) examines their influence on the organization of protein swarms. To assess this effect, we determined co-citation frequencies between protein swarms in Supplementary Table S1 and 136 disease-associated mature microRNAs in Medline abstracts. The result of the hierarchical clustering based on these collected co-citation frequency measurements are presented in Figure 6.

Using a confidence threshold in cluster similarity values greater than 0.897 to separate protein swarm associations shown in Figure 6, we identified 20 clusters (PSA **1–20** in Table 3) with swarm compositions previously described in Figure 3. Similarly, applying a confidence threshold in cluster similarity values above 0.67 for grouping microRNAs resulted in 23 clusters, as shown in Table 3.

**Table 3:** List of microRNA clusters associated with protein swarm associations 1–20.

Cluster identifier	microRNA within the cluster	Cluster identifier	microRNA within the cluster
A	mir-125b-5p, mir-138, mir-451a	M	**mir-26a, mir-29a**
B	**mir-143, mir-17, mir-192, mir-421**	N	**mir-126, mir-92a**
C	mir-196, mir-618	O	**mir-200c, mir-203**
D	mir-125a-3p, mir-369	P	mir-181b, mir-326
E	mir-1207, mir-936	Q	mir-133a, mir-423
F	mir-1246, mir-183	R	mir-133b, mir-193a, mir-200a, mir-7
G	mir-425-5p, mir-212	S	mir-34a, mir-34c
H	mir-129, mir-298	T	mir-221, mir-222
I	mir-29b-2, mir-590	U	**mir-107, mir-21**
J	mir-106a, mir-483-5p	V	**mir-150, mir-223, mir-155,**
K	mir-208a, mir-483		**mir-142-3p**
L	**mir-520c, mir-100, mir-486,**	W	mir-146a, mir-146a-5p
	**mir-5100, mir-542**		

Bolded values represent microRNAs that play a key role in COVID-19, atherosclerosis, Alzheimer's disease.

**Figure 7:**
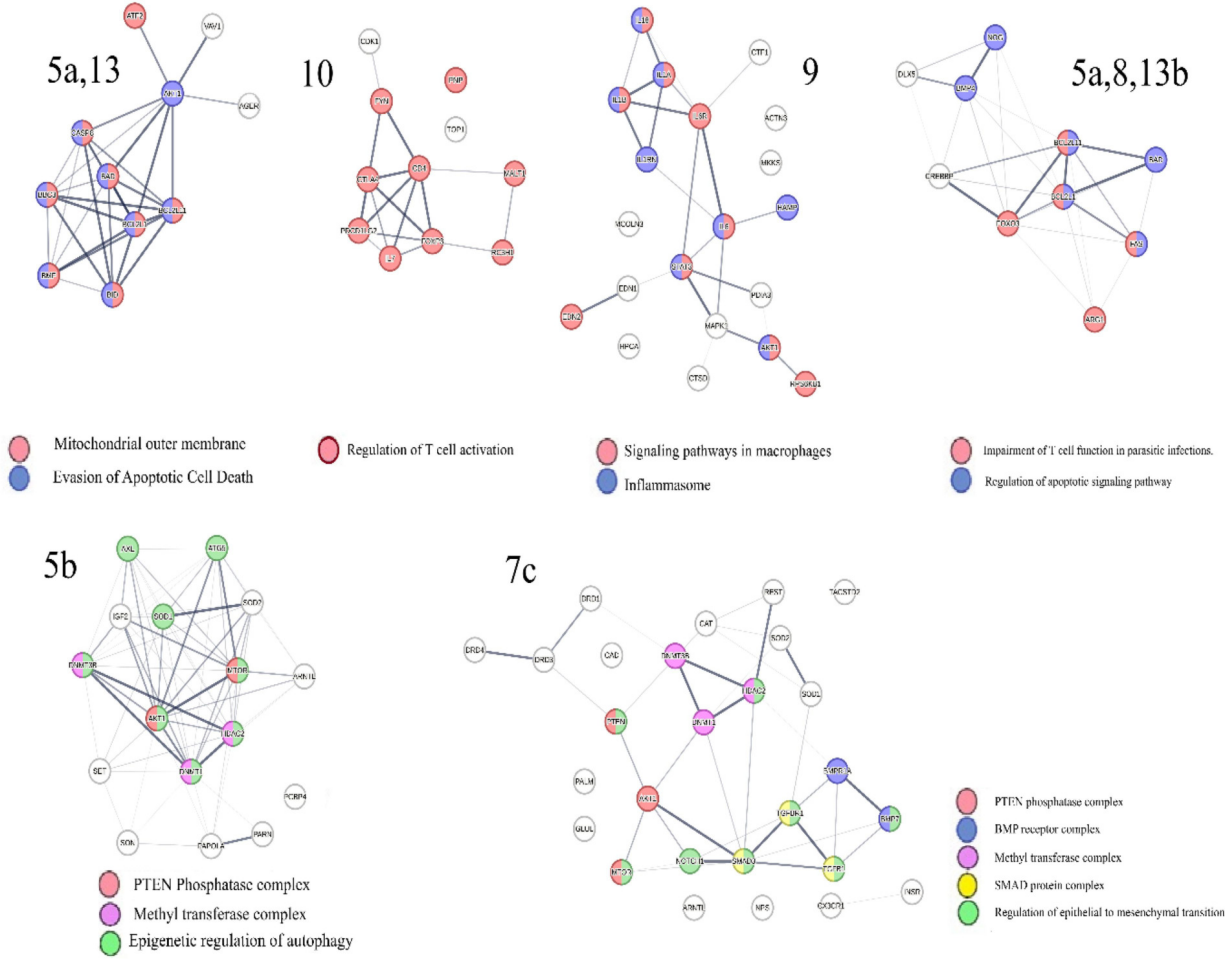
Identifies functions involving interactions between proteins in protein swarm associations (PSA) 5a,13 targeted by microRNAs clusters B, M, N, O and mir-195; **PSA 7c** targeted by microRNA cluster L, **PSA 9** targeted by microRNA cluster O; **PSA 10** targeted by microRNA cluster V and **PSA 5a,8 13b** targeted by microRNA cluster U; **PSA 5b** targeted by 61 microRNAs, **PSA 5a,13:** AGER, AKT1, ATF2, BAD, BBC3, BCL2L1, BCL2L11, BID, BMF, CASP8, VAV1; **PSA 7c:** AKT1, ARNTL, BMP7, BMPR1A, CAD, CAT, CX3CR1, DNMT1, DNMT3B, DRD1, DRD3, DRD4, GLUL, HDAC2, INSR, MTOR, NOTCH1, NPS, PALM, PTEN, REST, SMAD3, SOD1, SOD2, TACSTD2, TGFB1, TGFBR1; **PSA 9**: ACTN3, AKT1, CTF1, CTSD, EDN1, EDN2, HAMP, HPCA, IL18, IL1A, IL1B, IL1RN, IL6, IL6R, MAPK1, MCOLN3, MKKS, PDIA3, RPS6KB1, STAT3;**PSA 5a,8,13b**:ARG1, BAD, BCL2L1, BCL2L11, BMP4, CREBBP, DLX5, FAS, FOXO3, NOG; **PSA 5b**: DNMT3B, DNMT1, PARN, SET, HDAC2, ATG5, AXL, MTOR, PCBP4, PAPOLA, ARNTL, AKT1, SON, IGF2, SOD2, SOD1; **PSA 10:** CD4, CDK1, CTLA4, FOXP3, FYN, IL7, MALT1, PDCD1LG2, PNP, RC3H1, TOP1.

Analysis of the co-citation frequency distribution in Figure 6 indicates that the microRNA clusters listed in Table 3 exhibit specific patterns within protein swarm associations (PSA) **1–20**. These findings indicate that microRNAs within these clusters may modulate the expression levels of proteins associated with network topologies in cooperation network segments PSA **1–20**. Data suggesting potential cause-effect relationships associated with variations in microRNA expression and network connectivity are presented in Figure 7, which outlines the connectivity and functions of protein networks captured in PSA segments (**5a,13**), (**5b),** (**5a**,**8,13b**), (**7c**), (**9**), and (**10**) in Figure 6. Identification of these cause-effect relationships may also identify new strategies for developing effective disease treatments.

For example, protein network PSA (**5a,13**) in Figure 7 consists of proteins located in the outer mitochondrial membrane that regulate apoptosis via BH3-only proteins Bim (164). Network overlap analysis reveals that proteins in PSA (**5a,13**) are involved in cancer (165), infections (166–169), Alzheimer's disease (170, 171), and atherosclerosis (172). The presence of regulatory relationships between PSA (**5a,13**) and RNA expression levels in microRNA cluster **O**, which target expression levels of proteins in PSA (**5a,13**) is supported by observations showing that mir-195 (173–175), mir-200c (176–178), and mir-203 (179, 180) in microRNA cluster **O** regulate functions of BH3-only proteins and modulate activation and inhibition of apoptosis. This observation also suggests that identifying these cause-effect relationships may also identify new strategies for developing effective disease treatments for cancer, infections, Alzheimer's disease and atherosclerosis.

Similarly, proteins in cooperation network PSA (**7c**), which are targeted by microRNAs in Cluster L and mir-126, (associated with atherosclerosis (181, 182), and Alzheimer's disease, are members of a plasma membrane signaling receptor complex involved in the regulation of epithelial to mesenchymal transition (EMT) (183, 184). EMT is upregulated in people with Alzheimer's disease (185), and accelerates plaque growth and instability in atherosclerosis (186). Evidence for coregulation of functions within cooperation network PSA (**7c**) by microRNAs in cluster **L**, includes findings that mir-520c (187, 188), mir-100 (189), mir-486 (190), mir-5100 (191, 192), and mir-542 (193), in Cluster **L**, and mir 126 (189) regulate EMT and plasma levels of these microRNAs are associated with the severity of atherosclerosis (194–196), and Alzheimer's disease (197–199). These cause-effect relationships can also be used to identify new strategies for developing potentially selective disease treatments for atherosclerosis (194–196), and Alzheimer's disease.

Similarly, analysis of information related to proteins in PSA (**9**), which are targeted by microRNAs within cluster **O**, demonstrates that these proteins form part of the NF-kappa B complex and play key roles in modulating inflammation and macrophage polarization (200). The notion that microRNAs in cluster **O** co-regulate PSA **(9)** functions of is substantiated by the evidence indicating that mir-200c (201–204), and mir-203 (205–207), both in cluster **O** influence macrophage polarization and inflammatory processes via targeting NF-kappaB pathways. Furthermore, findings show that mir-200c (208, 209), and mir-203 (188), impact microglia polarization (210), and neuroinflammation, suggesting that the regulatory scheme captured in PSA **(9)** may also contribute to Alzheimer's disease (211, 212), and other dementias (213). Associations between mir-200c and mir-203 levels and the severity of coronary artery stenosis provide further evidence of the relevance of the PSA **(9)** regulatory scheme in atherosclerosis. These data suggest that the cause-effect relationships between PSA **(9)** and microRNAs within cluster **O** could be used for developing treatment options with a broader impact targeting inflammatory diseases, neuro-inflammation, dementia, and cardiovascular diseases.

Similarly, the retrieval of information associated with proteins in PSA (**10**) targeted by microRNAs in cluster **V**, demonstrates that these proteins play a role in regulating T cell activation, thereby bridging innate and adaptive immunity (214, 215). The hypothesis that microRNAs in cluster **V** co-regulate the function of PSA (**10**) is supported by findings indicating that mir-150, mir-223, mir-155 and mir-142 within this cluster are involved in T cell differentiation and in mediating interactions between innate and adaptive immune responses (216–220). Network overlap analysis of PSA (**10**) functions reveals that these mechanisms are exploited during immune evasion against parasites, cancer (221), and are also associated with the generation of pro-inflammatory T cell subsets in Alzheimer's disease (222), and atherosclerosis (223). Hence, the cause-effect relationships between PSA (**10**) and microRNAs within cluster **V** will have a substantial impact for seeking new approaches to target inflammatory disorders that are critical for immune health, protection against infections, as well as other disorders like cancer, Alzheimer's disease and atherosclerosis.

The regulation of T cell functions is further mediated by proteins in PSA (**5a,8,13b)**, including Arginase, which is recognized as a prominent swarm member and known to be involved in the regulation of T cell activation (224). The expression levels of proteins in PSA (**5a,8,13b**) are influenced by microRNAs in cluster **U**. Evidence for co-regulatory roles of mir-21and mir-107 in cluster U is provided by experimental observations showing that these microRNAs increase Arginase 1 expression (225, 226), exhibit immunosuppressive properties (227, 228), and modulate both T-cell activation (229, 230), and T cell apoptosis (231–233). Thus, interactions between PSA (**5a,8,13b)** and microRNAs in cluster **U** could be particularly useful for treatment of multiple disorders due to aberrant regulation of T-cell functions.

Additional evidence suggesting that PSA (**5a,8,13b)** acts as a regulatory network in atherosclerosis is the finding that deficiency of mir-21 in cluster **U** induces vascular inflammation during atherogenesis (136), while mir-107, also in cluster **U** enhances the repair of vascular endothelial cells (234). The involvement of these protective microRNAs in Alzheimer's disease is indicated by reports demonstrating reduced levels of mir-107 and mir-21in affected individuals (235, 236). These observations broaden the value of targeting cause-effect relationships between PSA (**5a,8,13b)** and microRNAs in cluster **U** for treatment of atherosclerosis as well as Alzheimer's disease.

Retrieval of information related to proteins in PSA (**5b**) indicates that these proteins participate in the epigenetic regulation of autophagy, which is key for regulating immune system functions (237, 238). Levels of protein expression within the cooperation network, PSA (**5b**) are influenced by 61 out of 136 disease-associated microRNAs, which includes 13 microRNAs linked to Alzheimer's disease, and 6 microRNAs linked to atherosclerosis (239), as well as mir-126 and mir-92a in micro–RNA Cluster **N**. Evidence suggests that these microRNAs coregulate functions within cooperation network PSA (**5b**) as both miR-126 and miR-92a have been implicated in regulating autophagy (240, 241). Additionally, mir-let7a, which targets proteins in the cooperation network PSA (**5b**), has been shown to regulate autophagy in an Alzheimer's disease model (242) and plays a key role in cardiovascular disease (243). Since the topology dynamics of PSA (**5b**) is regulated by most of the disease-associated microRNAs, the interactions of proteins in this cooperation network represent a prime target for developing new therapies for the multiple diseases discussed in this paper.

**Figure 8:**
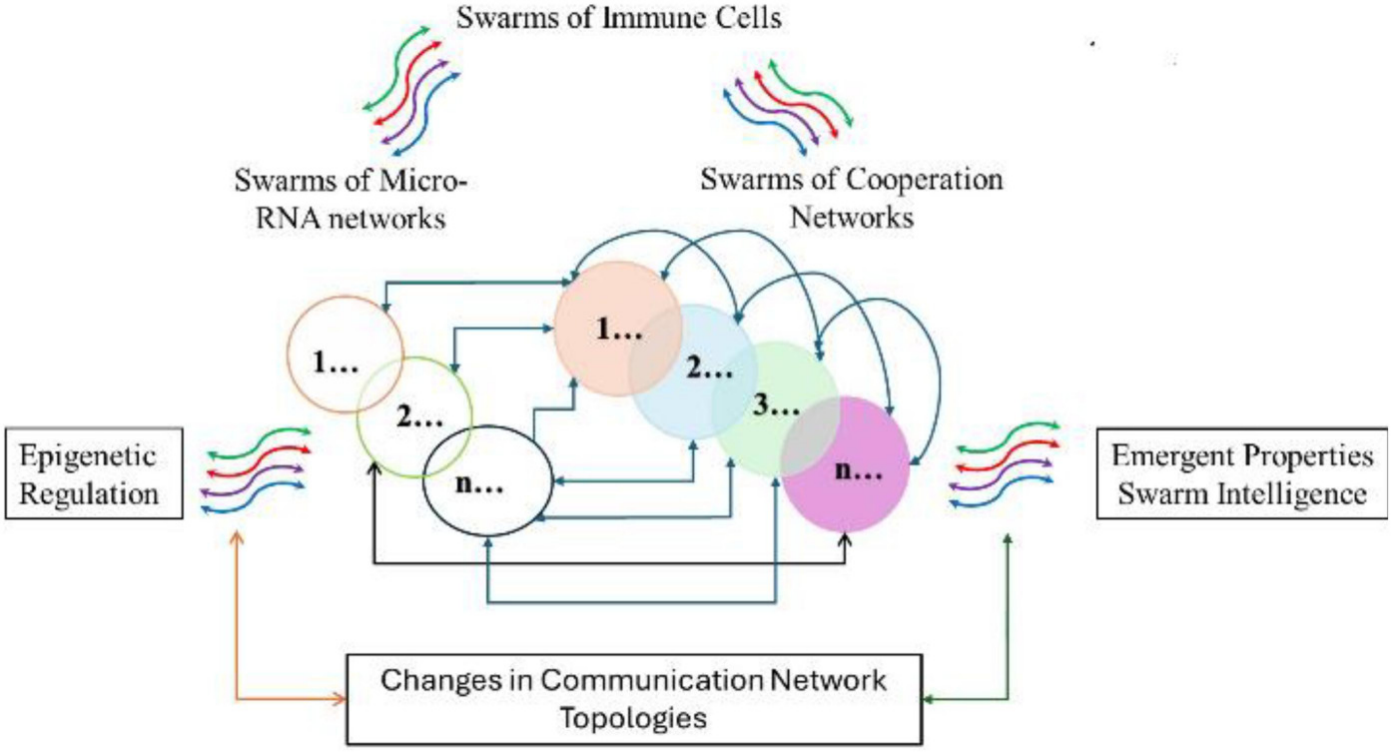
Illustrates an integrated regulatory system modulating functions of cooperation networks by modulating network topologies.

Overall, the cause-effect relationships summarized in Figure 7 imply that cooperation networks PSA **(1–20)** and microRNA clusters in Table 3 are parts of an integrated regulatory system illustrated in Figure 8, which controls the functions of networks coordinating information exchanges and interactions among protein complexes shown in Figure 5.

### Impacts of COVID-19 on cooperation networks PSA (**1–20**)

**Figure 9:**
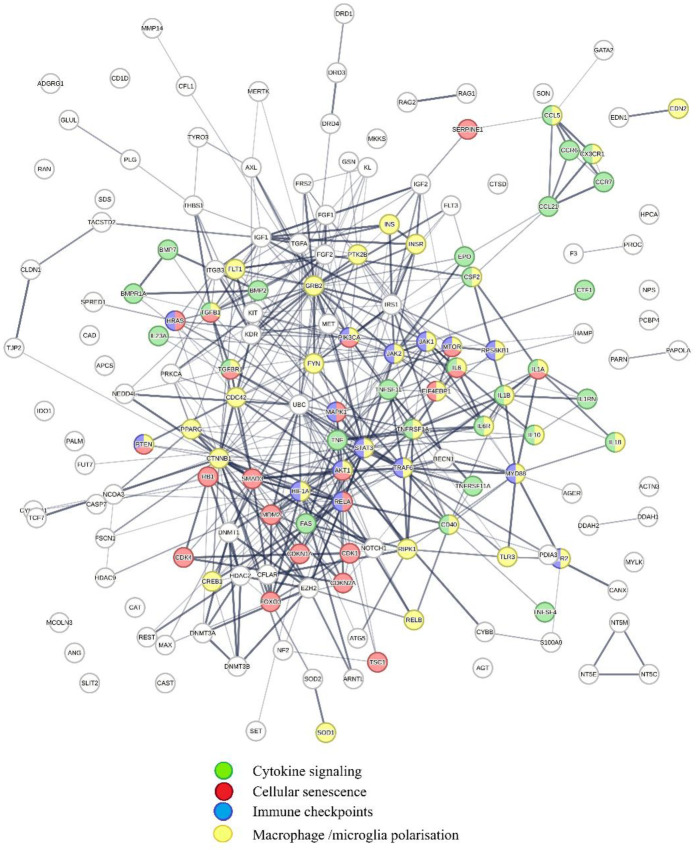
Shows physical interactions between proteins in PSA **1–20** targeted by COVID-19-associated microRNAs. Key functions affected by COVID-19 are cytokine signaling (green); cellular senescence (red); Immune checkpoints (blue); and macrophage/microglia polarization (yellow). The regulatory scheme highlighted shows that COVID-19 affects functions initiating and potentiating inflammation and regulating cellular senescence and immune checkpoints.

Impacts of COVID-19-induced perturbations on regulatory schemes captured in PSA (**1–20**) are summarized in Figure 9.

Retrieval of publications showing statistically significant network overlaps with protein interaction network shown in Figure 9 reveals that COVID-19-induced perturbations of cooperation networks captured in PSA (**1–20**) influence diseases such as cancer (244, 245), infectious diseases (246–249), metabolic diseases (250–254), cardiovascular diseases (255–257), inflammatory diseases, (258–262). Alzheimer's disease (263, 264), and aging (265, 266). Macrophages expressing ACE2 receptor and lectin CD169 can be infected by SARS-CoV-2 (267), and adopt a proinflammatory M1-like phenotype (268), which promotes both inflammation and viral replication by generating senescent cells, which secrete additional pro-inflammatory and pro-coagulation factors (269–274), and regulate immune checkpoints (275–278). Proinflammatory host responses are further amplified by SARS-CoV-2-associated spike protein, which increases M1 macrophage polarization (279).

A critical mechanism driving the amplification of inflammatory responses in COVID-19 is the dysregulation of TGF*β* pathways, regulated in part by cooperation networks PSA (**9**) and PSA (**7c**) (280, 281). leading to hyperinflammation (282, 284), increased cellular senescence (283, 284), microclot formation (285), acute respiratory distress syndrome (ARDS) (286), pulmonary fibrosis (287), and life-threatening sepsis (288, 289). TGF*β* signaling also lowers mir-125a-3p, mir-369 expression (cluster **D**; Table 3), which normally help regulate inflammation and TGF*β* pathways by targeting proteins in PSA (**9**) and PSA (**7c**). Thus, mir-125a-3p, mir-369, reduce the expression of ACE2 (290), leading to loss of anti-inflammatory effects associated with ACE2 expression (291).

In long COVID, persistent proinflammatory conditions can result from reactivation of dormant viruses such as Epstein–Barr and varicella zoster (292), shifting microglia polarization in the brain to proinflammatory states and causing hyperinflammation, brain fog, and cognitive dysfunction (105, 293). Dysregulation of cooperation networks PSA (**10**) and PSA (**5a, 8, 13b**), along with sustained proinflammatory macrophage/microglia polarization and further aggravated by circulating spike proteins (92, 294–297), impairs T cell functions (298), and disrupts the regulatory system (see Figure 8) that fine-tunes inflammation (299–305).

### Effects of treatments targeting the regulation of cooperation networks

The current methodology serves as an analytical tool for addressing a range of diseases. Analysis of complex relationships between microRNAs and cooperation networks identifies potential areas for the development of new treatment approaches. These strategies may complement existing drug discovery methods, as they can identify multiple disease targets (including proteins and microRNAs) and offer broader perspectives for understanding the mechanistic outcomes involved in diseases.

The summary of results presented in Table 4 introduces the concept of co-regulation of cooperation networks by microRNAs for developing therapeutic strategies relevant to the indications described in this manuscript. Additionally, it outlines approaches for examining the connections between different diseases and their mechanisms of action. For instance, the interaction between PSA (5a,8,13b) and cluster U can be used to identify specific microRNAs in cluster U that are either upregulated or downregulated in the target disease. Treatment options can then be determined through traditional screening methods by identifying substances that counteract the effects of disease-associated microRNA cooperation and protein interaction networks.

**Table 4:** Disease associations due to co-regulation of microRNAs with cooperation networks (PSAs).

Protein Swarm Associations (PSA)	MicroRNA cluster	COVID-19 & other infections	Atherosclerosis & cardiometabolic diseases	Alzheimer's disease & dementias	Inflammation & immunity	Other
5a.13	O	+	+	+		Cancer, regulation of apoptosis
7c	L/mir-126	+	+	+		Epithelial to mesenchymal transition
9	O	+	+	+	+	Macrophage/microglial polarization
10	V	+	+	+	+	Cancer
5a,8,13b	U	+	+	+	+	Aberrant regulation of T-cell functions
5b	Interacts with 61/136 microRNAs	+	+	+	+	Aging (PSA5b is master coregulator of autophagy)
	N	+	+	+		Aging/autophagy
	Mir let7a	+	+	+		Aging/autophagy
I-20	is targeted by COVID-19-associated microRNAs	+	+	+	+	Cancer, coagulopathy, aging

Understanding these relationships supports the development of new treatment strategies, such as combinations of arginine, hesperidin, quercetin, vitamin D, and zinc for respiratory and immune disorders (see Figure 9).

Vitamin D was selected as a starting point for targeting epigenetic regulation of the regulatory scheme (see Figure 8) due to its broad effects on immune system modulation (306–308). Co-citation frequency measurements associated with Vitamin D deficiency (Figure 3) showed that vitamin D deficiency affects functions of multiple cooperation networks **1, 4, 5a, 5b, 13a, 7b, 7c, 8, 9, 7d, 14, 10, 11** and **12,** many of which are regulated by microRNAs such as mir-155, mir-146a, mir-34a, and mir-21 (see Figures 3, 5B, 6, 8). SARS-CoV-2 infection increases the expression of the microRNAs mir-155, mir-146a (309), mir-34a (310), mir-21 (311), while Vitamin D supplementation reduces levels of mir-155 (312), mir-146a (313), mir-34a (314), and mir-21 (315). And has been shown in clinical trials to decrease COVID-19 severity (316). Figure 8 shows the influence of COVID-19-associated microRNAs on immune functions, suggesting that the induction of cellular senescence plays a key role in COVID-19 pathology (284, 317). The impact of cellular senescence on microRNA expression levels is provided by observations showing that the induction of cellular senescence increases the expression levels of pro-inflammatory mir-155 (318), mir-146a (319), mir-34a (320, 321), and mir-21 (322).

Counteracting effects of cellular senescence on microRNA expression levels, senolytic flavonoids such as quercetin (323) and apigenin (324), downregulate cellular senescence (325, 326), and, in doing so, decrease the expression levels of mir-155 (327, 328), mir 146a (329, 330), mir 34a (331, 340), and mir-21 (332). Benefits resulting from this senolytic action is suggested by observations showing that quercetin decreases the severity of COVID-19-induced inflammation and expedites recovery (333), and that apigenin deactivates the NLRP3 inflammasome (334).

COVID-19 is linked to arginine deprivation (335), and dysregulation of T-cell functions (335–337). Figure 7 shows that networks PSA (**5a, 8, 13b)** which regulate T-cell functions by regulating Arginase 1, are targeted by mir-107 and mir-21 in microRNA cluster **U** (Figure 6). Arginine deficiency increases mir-21 expression and inflammation by lowering endothelial nitric oxide production (338). Arginine supplementation increases mir-21 expression levels in clinical studies and shown to reduce the duration of in-hospital stays and the need for respiratory support in COVID-19 patients (339).

Zinc deficiency is associated with increased pro-inflammatory mir-21 and impaired T-cell function; supplementing zinc restores balance, lowers mortality, and shortens hospitalizations. Chronic inflammation and pulmonary fibrosis after COVID-19 are tied to dysregulation of networks 9 and 7c, leading to excessive TGF*β*1 signaling and decreased mir-132. Hesperidin increases mir-132 and inhibits TGF*β*1, improving immunity and controlling cytokine storms.

Zinc deficiency has also been correlated with persistent inflammation and increased mortality in COVID-19 patients. Zinc deficiency leads to overexpression of proinflammatory mir-21 (340–342). and to impair T cell functions (343, 344); zinc supplementation reverses mir-21 imbalance (345), and has been reported to lower 30-day mortality and shorten hospitalizations of COVID-19 patients (346, 347) Some of the major long-term problems associated with COVID-19 are the development of chronic inflammation (348) and pulmonary fibrosis (349). These conditions are precipitated in part by COVID-19-induced dysregulation of cooperation networks **9** and **7c**, leading to excessive TGF*β*1 signaling (350, 351) and decreases in mir-132 levels functioning as a suppressor of pro-inflammatory cytokine production (352, 353). Counteracting effects produced by excessive TGF*β*1 signaling is the flavonoid hesperidin; it increases mir-132 levels (354, 355), and inhibits TGF*β*1 activity (356). Benefits resulting from these mechanisms of action are provided by observations showing that hesperidin supplementation improves immunity against infections and controls cytokine storms (357).

Targeting the same regulatory schemes are combinations of Vitamin D and hesperidin in anti-aging studies (358), and the use of arginine and zinc combinations to improve thymic endocrine activity and peripheral immune functions in aged mice (359). The results indicate that clinical trials evaluating combinations of Vitamin D, arginine, quercetin, hesperidin, and zinc may demonstrate advantages in anti-aging and immunological research, as well as potential positive outcomes in studies related to COVID-19 and associated conditions.

## Discussion

MicroRNAs are integral to various biological processes and serve as important biomarkers and potential therapeutic targets (360). To enhance the use of microRNA data for developing new avenues for disease treatment, including cardiovascular disease and cancer, bioinformatics tools have been applied to analyze microRNA expression profiles and functional roles (361–366). Nevertheless, the intricate nature of network-network interactions and the regulatory effects of microRNAs on protein networks remain largely uncharacterized, with current understanding primarily derived from theoretical bioinformatic analyses rather than direct cause-effect relationship studies (367). For instance, in efforts to elucidate the role of microRNAs in the pathogenesis of diabetic cardiomyopathy, a machine learning tool called DeepMiRBP has been used for predicting microRNA-protein network interactions (368). Additionally, extensive literature searches using multiple electronic databases such as PubMed, Scopus, Web of Science, and Google Scholar have helped examine the complex association between microRNAs and oxidative stress in cardiovascular disease (369). To identify the role of key microRNAs and mRNAs related to inflammatory bowel diseases (IBDs) and their subtypes, text mining-based approaches leveraging PubMed and PMC databases have been employed (370). Similarly, a web-based Random Walk algorithm (371) has been introduced to explore linkages between microRNA expression levels and disease-associated pathways. Furthermore, deep learning models specifically designed to predict microRNA-binding proteins have been proposed by simulating molecular interactions (372).

In contrast, the findings from our protein swarm-based cause-effect analysis, as depicted in Figures 2–9, demonstrate that interactions within the microRNA protein network modulate the precision and specificity of biological mechanisms/machinery coordinating cellular cooperation and processes across all systems levels (299, 373, 374). Examination of protein swarm organization in Figures 3, 6 provides insights into how regulatory intelligence differentiates health from disease, highlighting the influence of diseases and microRNAs on the structural regulation of cooperative functions within these networks (375).

Notably, the strong cluster similarity metrics integrating proteomics data in Figures 3, 6 suggest overlapping topologies among cooperation network segments **1–20**. Figure 2 substantiates this premise by revealing that proteins within these network segments are capable of physically interacting with proteins across all 20 network segments. Additionally, Figure 5B indicates that these proteins contribute not only to information transmission but also to the execution of specific cellular functions (376, 377). These network characteristics facilitate efficient information dissemination and the coordination of cooperation among diverse functional processes. Furthermore, alterations in microRNA expression levels (378–380) can increase or decrease the number and the strength of interactions within protein networks, thereby reshaping network topology and enabling epigenetic regulation for dynamic adjustments and precise modulation of cooperation network functions.

The observed cause-effect relationships illustrated in Figures 2–9 collectively show that perturbations of microRNAs expression influence macrophage plasticity and the progression and severity of various diseases, including cardiovascular disease (381).

The section entitled “*Effects of treatments targeting the regulation of cooperation networks*” builds upon previous research by illustrating that integrating vitamin D's genomic and non-genomic signaling—through the coupling of nitric oxide and redox signaling—may improve the efficacy of vitamin D-based therapies for cardiovascular disease (382). Additionally, protein swarm-based, cause-effect analysis enables evaluation of treatment effects on cooperative networks via combinations such as vitamin D with quercetin, hesperidin, arginine, and zinc ascorbate, which are designed to modify disease-specific expression. This approach shows potential not only for cardiovascular diseases but also for other disorders linked to dysfunctional cooperation networks (383, 384).

Ultimately, findings from protein swarm-based cause-effect analyses support the advancement of therapies focusing on the topological architecture of cooperative networks, as opposed to targeting isolated pathways. This approach has proven effective in various contexts, including combination cancer therapies (385, 386), modulation of microbiome compositions for cardiovascular disease (387), and practices rooted in traditional medicine (388, 389).

## Strengths and limitations

Understanding the influence of protein network–protein network and microRNA network–protein network interactions on health and disease is essential for developing safe and effective therapeutic strategies (390). Our findings indicate that approaches focusing solely on individual components are insufficient to capture the emergent properties resulting from dynamic network-network interactions that affect disease progression and treatment outcomes (391). To address this complexity, protein swarm-based cause–effect analysis utilizes the inherent plasticity of proteins, providing an unbiased framework to examine how emergent properties within interacting network systems modulate physiological and pathological states of organisms (392). Applying “Swarm Intelligence” as an analysis tool enables the delineation of ultra-complex cause-effect relationships that are otherwise challenging to ascertain and resolve (393).

Nevertheless, current cause–effect analyses are constrained by their predominant focus on the effects of mature microRNAs within cooperative systems, while frequently neglecting the role of post-translational modifications in proteomes. Furthermore, technical constraints restrict the resolution of studies on network–network interactions due to upper bounds on the number of protein swarms that can be sampled from tissue proteomes. Finally, the effectiveness of machine learning approaches in addressing complex biological cause–effect relationships remains constrained by insufficient data regarding concentration and temporal dependencies on a system-wide scale (413).

Moreover, methodological limitations impose constraints on the resolution of studies examining network–network interactions, as there are upper limits to the number of protein groups that can be sampled from tissue proteomes. Additionally, while machine learning approaches offer promise in elucidating complex biological cause–effect relationships, their effectiveness is currently limited by insufficient data describing concentration and temporal dependencies at the system-wide level.

## Summary

Protein swarm-based cause-effect analysis was employed to investigate the principles governing immune system responses to injury, emphasizing dynamic changes in protein network topologies and the epigenetic regulation of microRNAs. These mechanisms function as computational systems that facilitate rapid adaptation to fluctuations in both micro and macro environments. Disruptions affecting the accuracy of protein network topology-mediated computations offer insights into the immune evasion strategies of pathogens (261, 394), and cancers (395–397), and parallels in pathological processes among infections (398), Alzheimer's disease (399–404), metabolic disorders (405, 406), cardiovascular diseases (407), and autoimmune diseases (408). Furthermore, deciphering complex cause-effect relationships arising from emergent computations (409–411), indicates that swarm-based cause-effect analysis may have applications not only in drug design but also in robotics and quantum computing (412, 413).

## Data Availability

The datasets presented in this study can be found in online repositories. The names of the repository/repositories and accession number(s) can be found in the article/Supplementary Material.
